# Cold Atmospheric Plasma: A Promising Controller of Cancer Cell States

**DOI:** 10.3390/cancers12113360

**Published:** 2020-11-13

**Authors:** Xiaofeng Dai, Kateryna Bazaka, Erik W. Thompson, Kostya (Ken) Ostrikov

**Affiliations:** 1 Wuxi School of Medicine, Jiangnan University, Wuxi 214122, China; 2 Wuhan Ammunition Life-Tech Company, Ltd., Wuhan 430200, China; 3 Hospital of Xi’an Jiaotong University, Xi’an 710061, China; 4 Research School of Electrical, Energy and Materials Engineering, College of Engineering and Computer Science, The Australian National University, Canberra, ACT 2600, Australia; Katia.Bazaka@anu.edu.au; 5 Institute of Health and Biomedical Innovation, Queensland University of Technology, Brisbane, QLD 4059, Australia; e2.thompson@qut.edu.au (E.W.T.); kostya.ostrikov@qut.edu.au (K.O.); 6 School of Biomedical Sciences, Queensland University of Technology, Brisbane, QLD 4059, Australia; 7 Translational Research Institute, Woolloongabba, QLD 4102, Australia; 8 School of Chemistry and Physics, Queensland University of Technology, Brisbane, QLD 4000, Australia

**Keywords:** cold atmospheric plasma, cancer state transition, reactive species, oncotherapy

## Abstract

**Simple Summary:**

Cancer treatment is complicated by the distinct phenotypic attractor states in which cancer cells exist within individual tumors, and inherent plasticity of cells in transiting between these states facilitates the acquisition of drug-resistant and more stem cell-like phenotypes in cancer cells. Controlling these crucial transition switches is therefore critical for the long-term success of any cancer therapy. This paper highlights the most promising avenues for controlling cancer state transition events by cold atmospheric plasma (CAP) to enable the development of efficient tools for cancer prevention and management. The key switches in carcinogenesis can be used to halt or reverse cancer progression, and understanding how CAP can modulate these processes is critical for the development of CAP-based strategies for cancer prevention, detection and effective treatment.

**Abstract:**

Rich in reactive oxygen and nitrogen species, cold atmospheric plasma has been shown to effectively control events critical to cancer progression; selectively inducing apoptosis, reducing tumor volume and vasculature, and halting metastasis by taking advantage of, e.g., synergies between hydrogen peroxide and nitrites. This paper discusses the efficacy, safety and administration of cold atmospheric plasma treatment as a potential tool against cancers, with a focus on the mechanisms by which cold atmospheric plasma may affect critical transitional switches that govern tumorigenesis: the life/death control, tumor angiogenesis and epithelial–mesenchymal transition, and drug sensitivity spectrum. We introduce the possibility of modeling cell transitions between the normal and cancerous states using cold atmospheric plasma as a novel research avenue to enhance our understanding of plasma-aided control of oncogenesis.

## 1. Introduction

Reactive oxygen and nitrogen species (RONS), being either internally produced from cell metabolism or externally introduced on environmental exposure, could directly modulate the environment of cells and affect their behavior [1]. Cold atmospheric plasma (CAP) has rapidly emerged as a highly effective means for the generation and delivery of controlled doses of reactive species to cancer cells in vitro and in vivo in the last 5 years. Although proven effective in evoking oxidative stress-induced cell toxicity and death, CAP was not initially designed to be used as a source of reactive species to selectively target events that may induce, halt or reverse cancer progression. Instead, CAP was originally developed for material synthesis and processing, and the early uses of CAP in the medical field centered around surface decontamination of biomaterials. Recent advances in plasma technology and diagnostics methods have overcome some of the challenges associated with the generation and targeted delivery of highly controlled combinations of RONS to cancer cells to effectively control events critical to cancer progression. These include potently and selectively inducing apoptosis, reducing tumor volume and vasculature, and halting metastasis taking advantages of, e.g., synergies between long-lived species H${}_{2}$O${}_{2}$ and nitrite that are delivered from CAP [2,3,4]. As it is an emerging field, medical use of CAP as a standalone or adjuvant cancer therapy is at the very early stages of exploration. The first clinical trial where CAP was used as an oncotherapy was approved by FDA in mid-2019. The trial aimed to save the life of a 33-year old patient with a relapsed incurable peritoneal sarcoma patient and prolong the life span of a 75-year old patient carrying late stage pancreatic cancer for 2 years [5,6].

Despite a rapid increase in the number of promising reports that showcase CAP selectivity and efficacy against a wide range of cancer types in recent years [7,8,9,10,11], little is known about the roles of various plasma-generated physical and chemical effects in controlling core cancer state transitions, such as tumor life/death transition, angiogenic switch, tumor metastatic transition, and drug sensitivity spectrum. This paper aims to stimulate discussion and encourage the design of in vitro, ex vivo and in vivo studies that take advantage of unique features of CAP therapy for effective cancer management. To achieve this goal, we discuss what is known about the ability of CAP to modulate and control the critical molecular events driving cancer initiation and progression through generating reactive species. We then attempt to draw parallels between what is already known about the mechanisms of CAP activity, the broader literature about the effects of RONS (and to lesser extent, the electric fields and UV light) on tumorigenesis, and the knowledge we would need to develop in order to use CAP as a cancer state controller that relies on reactive species generation.

## 2. CAP Efficacy through Reactive Species

In contrast to other oncotherapies that generate reactive species, CAP relies on RONS generation as the primary inducer of specific tumorigenesis events. CAP could potentially enhance the selectivity and efficacy of targeting due to the flexibility and controllability of plasma-generated effects, even to the point of introducing a feedback loop for in situ modulation of reactive species chemistry and intensity in response to changes in the cell behavior [12]. This is a highly unique feature of CAP which when coupled with rapid and targeted delivery of highly reactive chemistries to the cells, highlights strong potential of this emerging oncotherapy. CAPs have very low degrees of ionization and low bulk gas temperatures, which enable their direct application to living tissues [7]. The ionization process produces a rich assortment of biologically relevant species, including free radicals, electrons, ions, photons and reactive molecules [2,13]. Their chemistry, reactivity, and relative abundance are controlled by chemistry of the working gas, intensity and nature of the applied field, and proximity to targets, e.g., cells or media. In addition to chemical species transported to cells from CAP, the interactions between plasma-generated physical effects with the molecules at the gas-liquid interface, and within the bulk liquid phase, can generate RONS, e.g., OH·, O, O${}_{2}$ (1Δ), O${}_{2}^{\cdot }$${}^{-}$, H${}_{2}$O${}_{2}$, and NO, within the treated media and intracellularly [14,15]. The physical effects in question include energetic electrons, photons, electric fields and shockwaves. These chemicals could interact with cell surface to achieve various cell responses. Examples include the preference of H${}_{2}$O${}_{2}$ in attacking the amino acids near heme group [16] and the requirement of interactions between short-lived species like singlet oxygen with catalase and NOX1 that are often present on tumor cell surface [2].

### 2.1. CAP Alters the Life/Death Transition

**Figure 1:**
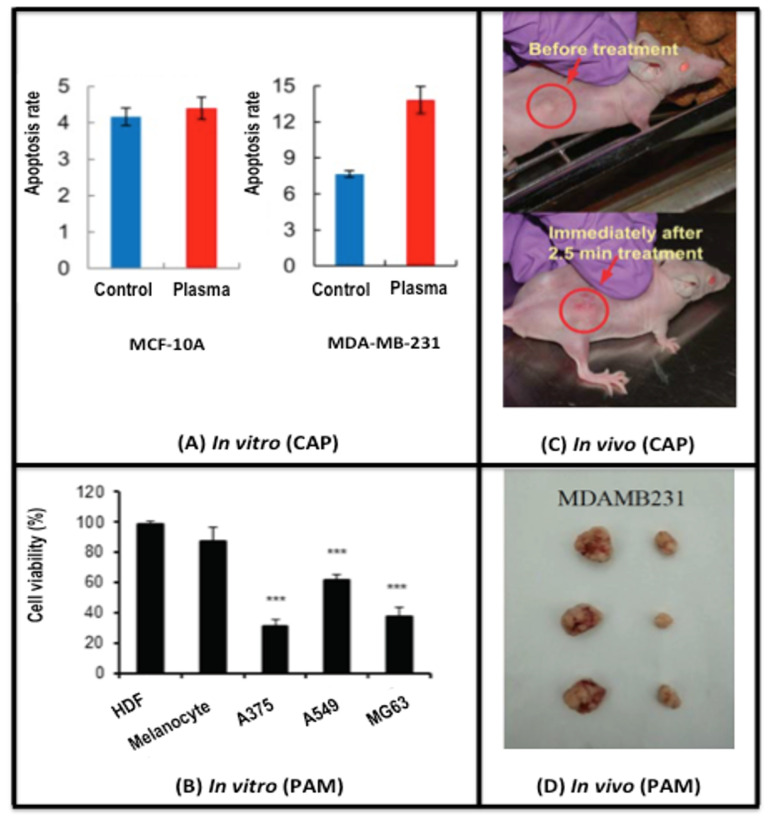
CAP-induced selective cancer cell death. (**A**) in vitro evidence showing the selectivity of CAP on cancer cells. The apoptosis rate of cancer cells (MDA-MB-231) increases 1.75-fold after CAP treatment, while that of non-transformed cells (MCF-10A) does not significantly change. Reproduced with permission [23]. Under the terms of the Creative Commons Attribution License. (**B**) in vitro evidence showing the selectivity of PAM on cancer cells. The cell viabilities after PAM treatment significantly decrease in cancer cells (A375, A549, MG63), but do not change with statistical significance in non-transformed cells (HDFs, melanocytes). Reproduced with permission [49]. Under the terms of the Creative Commons Attribution License. (**C**) An in vivo subcutaneous tumor model showing the efficacy of CAP in cancer shrinkage. The tumor of about 5 mm in diameter shrinks without observing any damage to the normal tissue immediately after 2.5 min single time CAP treatment. Reproduced with permission [22]. Under the terms of the Creative Commons Attribution-Noncommercial-Share Alike 3.0 Unported License. (**D**) An in vivo orthotopic tumor model showing the efficacy of PAM in cancer shrinkage. The tumor size was considerably reduced after injecting PAM into MDA-MB-231 cell-inoculated tumors of mice. The measurement was conducted at the 29th day after PAM injection. Reproduced with permission [50]. Copyright © 2018, Elsevier. PAM is short for ‘plasma-activated medium’. By listing evidence of both CAP and PAM in achieving therapeutic efficacy against cancer cells both in vitro and in vivo, we can see that it is indeed the RONS that play the most critical role in plasma cancer therapy as PAM lacks other effects of CAP such as fields, UV, etc. *** means *p* < 0.05 from student T test.

**Figure 2:**
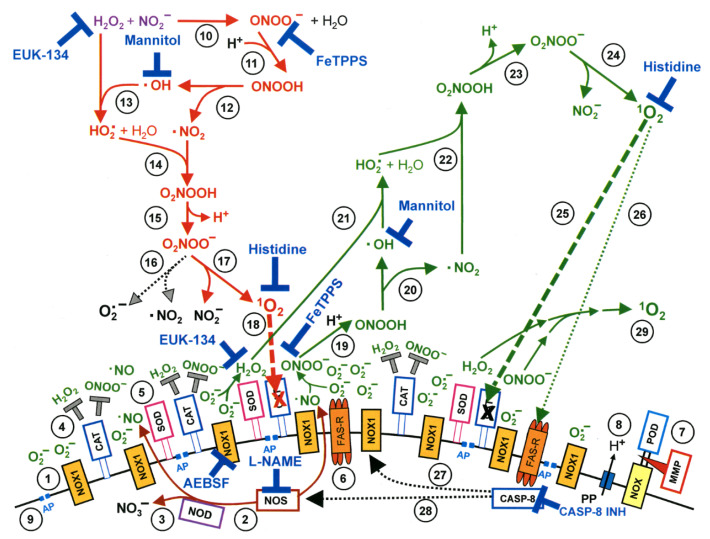
Interactions between CAP components and cell surface that trigger cell apoptosis through generating primary and secondary singlet oxygen (${}^{1}$O${}_{2}$). NADPH oxidase 1 (NOX1) is expressed in the membrane of tumor cells and generates extracellular superoxide anions (O${}_{2}^{\cdot }$${}^{-}$) (#1). NO synthase (NOS) (#2) generates ·NO which can be either oxidated by ·NO dioxygenase (NOD) (#3) or pass through the cell membrane. Membrane-associated catalase (#4) protects tumor cells towards intercellular RONS-mediated signaling. Comodulatory SOD (#5) is required to prevent O${}_{2}^{\cdot }$${}^{-}$-mediated inhibition of catalase. Further important elements in the membrane are the FAS receptor (#6), Dual oxidase (DUOX) (#7), from which a peroxidase domain (POD) is split through matrix metalloprotease, proton pumps (#8) and aquaporins (#9). H${}_{2}$O${}_{2}$ and NO${}_{2}^{-}$ derived from CAP treatment and stable in PAM interact and generate peroxynitrite (ONOO${}^{-}$) (#10). In the vicinity to membrane-associated proton pumps ONOO${}^{-}$ is protonated to peroxynitrous acid (ONOOH) (#11) and decomposes into ·NO${}_{2}$ and ·OH radicals (#12). ·OH radicals react with H${}_{2}$O${}_{2}$, resulting in the formation of hydroyperoxyl radicals (HO${}_{2}\cdot$) (#13). The subsequent generation of peroxynitric acid (O${}_{2}$NOOH) (#14) and peroxynitrate (O${}_{2}$NOO${}^{-}$) (#15) allows for the generation of “primary singlet oxygen” (${}^{1}$O${}_{2}$) (#17). Primary ${}^{1}$O${}_{2}$ causes local inactivation of membrane-associated catalase (#18). Surviving H${}_{2}$O${}_{2}$ and ONOO${}^{-}$ at the site of inactivated catalase are the source for sustained generation of “secondary ${}^{1}$O${}_{2}$” through reactions #19–#24. Secondary ${}^{1}$O${}_{2}$ may either inactivate further catalase molecules (#25) and thus trigger auto-amplification of ${}^{1}$O${}_{2}$ generation (#29), or activate the FAS receptor (#26) and in this way enhance the activities of NOX1 and NOS. This enhances the efficiency of secondary ${}^{1}$O${}_{2}$ generation. The site of action of specific inhibitors and scavengers are indicated. This figure was obtained with permission from [13] under the terms of Creative Commons CC BY license.

CAP alters the life/death transition via selectively killing cancer cells in a dose-dependent manner, i.e., from growth arrest to apoptosis and to necrosis as CAP doses increase [17,18]. The dose can be defined, e.g., by the amount and effectiveness of synergies created between H${}_{2}$O${}_{2}$ and nitrites. These synergies depend on both the concentration and proportion of CAP components as well as cell surface characteristics [2,3,4]. Such a process involves CAP-induced metabolic stress with altered metabolite profiling [19] and a modified tumor microenvironment [20], and involves mostly epigenetic [21] alterations. Pre-clinical evidence shows selective cytotoxicity of CAP against malignant rather than normal cells [6,22,23,24,25] (Figure 1). This selectivity has been attributed to more easily tilted balance between pro- and anti-oxidants in early-stage cancer cells as compared with normal cells, with cancer cells with a higher baseline RONS level due to the elevated rate of RONS creation and turnover than normal cells. Once the RONS concentration is raised in neoplastic cells due to endogenous perturbations, they have no excess anti-oxidant capacity and are forced to undergo apoptosis. In contrast, normal cells can keep their RONS level from reaching the apoptosis-inducing threshold using anti-oxidants in reserve [24]. For late stage malignant cells that had undergone tumor progression, they are protected against exogenous RONS stress by membrane-associated catalase; singlet oxygen mediated catalase deactivation occurs prior to and is critical to H${}_{2}$O${}_{2}$ influx towards cellular redox regulation (Figure 2) [2,3,4,13]. Thus, although the uptake by all cells is similar, healthy cells can deal with the extra stress and cancer cells cannot. Such a process is induced by oxidative stress via the Srx-Nrf2 anti-oxidant system in colorectal cancer cells [26], and involves sestrin2-mediated nitric oxide synthase signaling in melanoma cells [27]. Ozone was implicated as another key player besides singlet oxygen and H${}_{2}$O${}_{2}$ in the underlying mechanism of CAP-mediated cancer cell apoptosis [28]. Increased peroxiredoxin expression of human osteosarcoma cells after CAP treatment supports the influence of CAP on cellular redox homeostasis [29]. A specific CpG site hypomethylation was uncovered in MDA-MB-231 breast cancer cells, representing CAP-induced epigenetic alterations [22]. Other potential mechanisms leading to CAP selectivity on cancer cells include characteristic expression of NOX1, catalase and SOD in advanced malignant cells that require catalase deactivation prior to RONS-induced lipid peroxidation [3,30]. Over-represented aquaporins on cancer cell cytoplasmic membranes mediating RONS entry [31], and low cholesterol fraction in cancer cell membranes that promotes RONS permission [32]. Also, tumor cells are more sensitive to endogenous H${}_{2}$O${}_{2}$ and more resistant to exogenous H2O2 than non-malignant cells. This is because tumor cells have a high local concentration of catalase on cell surface that protects them against exogenous H${}_{2}$O${}_{2}$ and peroxynitrite [33,34]. Singlet oxygen generated from a complex interaction between H${}_{2}$O${}_{2}$ and peroxynitrite triggers the generation of high concentrations of secondary singlet oxygen in tumor cells. The singlet oxygen inactivates the protective catalase in cells, promotes aquaporin-mediated influx of extracellular H${}_{2}$O${}_{2}$ and reactivates intercellular RONS-mediated apoptosis signaling [35,36]. Recently, CAP was demonstrated to be capable of inducing immunogenic cell death (ICD) in melanoma cells [37], A549 lung carcinoma cells [38] in vitro and in murine CT26 colorectal tumors in vivo [39]. Here the short-lived reactive species such as hydroxyl radicals, atomic oxygen and nitric oxide were the relevant active ingredients [36]. H${}_{2}$O${}_{2}$ was the central player in oxidant-induced apoptosis [40,41] but not in ICD [39]. CAP could also induce cell necrosis via transiently modifying the tumor microenvironment using normal primary human fibroblast cultures isolated from oral tissue [42]. Furthermore, CAP can trigger senescence via causing a calcium influx in melanoma cells [43], and induce immunogenic cell death in melanoma cells taking advantage of short-lived RONS [37]. It is worth emphasizing the importance of dosing on CAP’s selectivity against cancer cells that once exceeded the level that normal cells tolerate, can induce death events in non-malignant cells [44,45,46,47,48].

### 2.2. CAP Suppresses the Tumor Angiogenic Switch

The ability of CAP to selectively suppress VEGFA expression and induce that of 15 factors in osteosarcoma cells was shown through a RT2 Profiler PCR Array assay that detects 84 chemokines, growth factors, TNF superfamily members, interleukins, and cytokines. These results suggest a suppressive role of CAP on tumor angiogenic transition and altered tumor microenvironment [20]. Yet, on the other hand, CAP could activate angiogenesis-related molecules in normal cells such as skin keratinocytes, fibroblasts and endothelial cells for improved wound angiogenesis [51]. More efforts are required to investigate the effects of CAP on tumor angiogenesis given its relatively under-explored research status.

### 2.3. CAP Halts the Tumor Metastatic Transition

A CAP-induced halt on the cancer metastatic switch has been observed in cells of many human tumor types such as breast and colorectal cancers as well as melanomas [50,52,53]. CAP may affect metastasis by, e.g., regulating EMT (our own unpublished data), over-expressing MTSS1, a gene encoding the metastasis suppressor [53] and/or down-regulating MMP2/9, the expression or activity of which promotes metastasis [54,55]. Recent two studies using melanoma and pancreatic cancer cell models found no significant increase in EMT markers after CAP treatment, highlighting the need for more sophisticated test models for assessing the effect of CAP on EMT [56,57].

CAP may also convey selectivity on cancer stem cells (CSCs) as ROS production typically co-occurs with GSH extrusion [58]. The high concentration of the latter is one feature of less-differentiated cells including CSCs [59]. This is biologically plausible as CSCs have a relatively low redox level due to their stronger anti-oxidant capacity and CAP can modulate CSC plasticity by simultaneously reducing their anti-oxidant activities while elevating redox fluctuations.

### 2.4. CAP Modulates the Drug Sensitivity of Tumor Cells

CAP restores cell sensitivity to traditional therapeutic modalities [17], e.g., tamoxifen and temozolomide sensitivity in resistant MCF-7 breast cancer [60] and glioma [61] cells, respectively, and suppresses integrin signaling that mediates radio- and chemo-resistance in various types of cancer cells [62]. This has been attributed to a highly dynamic cocktail of activated and non-activated species uniquely associated with CAP, and potential synergistic effects that arise from the interplay between these species and plasma-generated physical effects. Tumor cells are highly heterogeneous in terms of susceptibility or resistance to different perturbations such as ionizing radiations and chemical species. This susceptibility or resistance can change during the treatment due to genetic or epigenetic alterations. As it is highly diverse in its chemical composition and biological activity, a plasma-generated cocktail of diverse particles and potential synergistic effects that arise from the interplay between these species and plasma-generated physical effects simultaneously target different biomolecules or pathways. These include the cellular anti-oxidant machinery in this heterogeneous cell population that constantly fluctuates between distinct states. Due to the multi-modality nature of CAP and the diversified targets that may evolve in tumors, the systematic rather than specific effects of CAP may restore the sensitivity of cancer cells to conventional approaches. Yet, these are more difficult to direct than canonical therapeutics.

## 3. CAP Safety

Free radicals produced by chemical and ionizing radiation (IR) tend to affect cell state transitions through direct interactions with cellular components. In contrast, CAP-generated singlet oxygen inactivates membrane-associated catalase that leads to extracellular auto-amplification of secondary singlet oxygen generation followed by substantial inactivation of membrane-associated catalase. The interactions of CAP with cell membrane lead to H${}_{2}$O${}_{2}$ influx and in parallel extracellular HOCl signaling that ultimately, result in lipid peroxidation by extracellular hydroxyl radicals (Figure 2) [2,3,63]. Such indirect contact between CAP and cellular component makes CAP a milder approach that offers a greater control over therapeutic efficacy, with less potential adverse effects as compared with chemical drugs and IR. However, while reactive species are generated outside of cancer cells, they can also diffuse into normal cells within the tumor microenvironment such as stroma cells and tumor-associated fibroblasts. Whether the increased redox level in these normal cells could induce altered signal transduction in the tumor microenvironment that ultimately affects cancer state transitions has not been explicitly studied and warrants thorough investigation. Clinical safety assessment of CAP has been conducted ex vivo on human skin, where a treatment of up to 2 min has been shown to safely avoid DNA damage [64]. Clinical testing of CAP efficacy and safety has been undertaken mostly as adjuvant treatment of chronic cutaneous ulcers in phase I or II clinical trials. CAP treatment was well tolerated in all cases, with no significant differences in adverse effect frequency observed [65].

## 4. CAP Administration Approaches

CAP can be generated directly using diverse plasma sources as well as administered indirectly to tumor cells in the form of plasma-activated medium (PAM).

### 4.1. CAP Generation Sources

Systems used for CAP generation are diverse, each producing a different configuration of physical effects and chemical species. From the application perspective, this provides therapeutic flexibility and a degree of control over desired cancer transitions compared with other oncotherapies. Typical configurations are a plasma jet and dielectric barrier discharge. Their efficacies have been tested in a plethora of human cancer types, such as breast, lung and colon tumors and melanoma in vitro, and their clinical efficacy was demonstrated in human advanced head and neck cancers [66]. Significant effort has led to an improvement in the efficacy of CAP devices and their suitability for clinical use, e.g., increasing penetration depth to 5 cm [63], and avoiding challenges of high voltage, intra-organic discharge formation, gas delivery and plasma probe volume [67]. In glioblastoma treatment in vitro and in vivo, a CAP device successfully delivered therapeutic doses of plasma across the blood-brain barrier with minimal damage to brain tissues [67].

**Figure 3:**
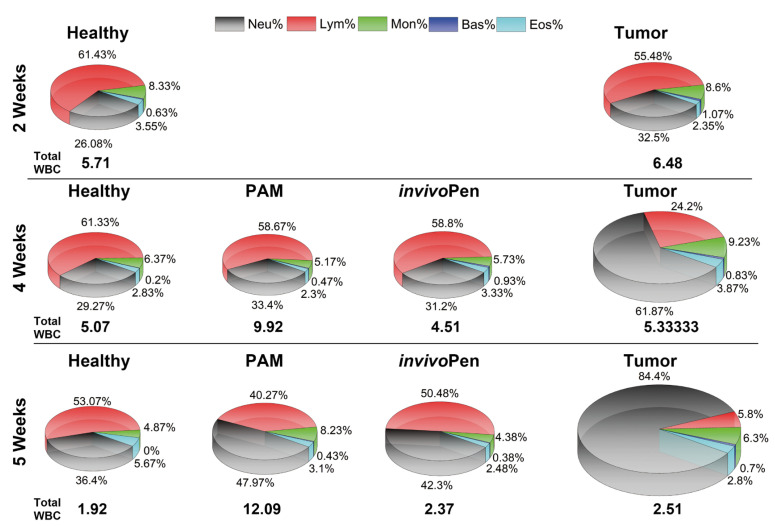
CAP-induced change on white blood cells. ‘Neu’, ‘Bas’, ‘Eos’, ‘Lym’, ‘Mon’, each is short for neutrophil, basophil, eosinophile, lymphocyte, and monocyte, respectively, which are measured components of white blood cells. ‘invivoPen’ and ‘PAM’ each represents direct and indirect treatment, respectively. Reproduced from [6] with permission. Copyright © the authors under the terms of the Creative Commons Attribution License.

PAM is an effective means for targeting tumors deep within the tissues, or for treatment of cancerous formation where exposure to physical effects, e.g., UV or mild heat, is not desired. Reactive species can be subcutaneously injected into tumors in a liquid form [25] or generated in situ through micron-sized sources such as an μ-plasma jet [63]. CAP can penetrate through tissue to the depth of approximately 50 μm [68] and up to 5cm to cause significant tumor growth recession in vivo [63]. Real skin is robust in blocking CAP-derived reactive specie such as OH, ${}^{1}$O${}_{2}$, O${}_{3}$ and H${}_{2}$O${}_{2}$ and allows limited penetration of certain types of RONS that are largely filtered out by the stratum corneum layers [69]. We had recently established a minimal invasive in situ plasma source, namely invivoPen, that could convey benefits over PAM in boosting animal immunity and reducing inflammation (Figure 3) [6]. Recent discoveries on plasma-induced ICD [37] largely extend our conception on the tumor region reached by CAP, where plasma-induced effects are rapidly carried over and transported by immune response cells throughout the body and into any tumor niche for eradicating malignant cell residues. To this extent, CAP can function as plasma pharmaceuticals alone or amplify the effects of existing onco-therapeutic modalities such as tamoxifen in the treatment of luminal breast cancers [60], and doxorubicin, epirubicin and oxaliplatin in sensitizing chemoresistant melanoma cells [70].

The diversity of these plasma sources and treatment modalities, however, makes in-depth elucidation of therapeutic mechanisms of CAP challenging. This is an area that is currently attracting significant attention from the rapidly growing plasma medicine community that spans physics, chemistry, biology, clinical medicine fields. Besides experimental advances, computational efforts modeling CAP-tissue interactions also help advance plasma biotechnology through, e.g., obtaining quantitative data on radical densities and their transformations in the liquid phase by including detailed physical and chemical processes at the interface for improved efficacy and accuracy [71].

### 4.2. CAP Activated Liquids

CAP can be administered indirectly to tumor cells in the liquid form such as PAM, plasma-activated PBS and plasma-activated saline [17,49,72,73,74,75,76,77,78]. In principle, plasma activation can be extended to any liquid form that is currently in use for cancer research, making it a more flexible tool for clinical use [79]. Yet, the selectivity of PAM against cancers is influenced by the medium to be activated. The selectivity of PAM is also more sensitive to influential factors such as cell type as compared with direct CAP treatment [80,81]. It was reported that PAM-induced cell death strongly depends on the combination of H${}_{2}$O${}_{2}$ and NO${}_{2}$${}^{-}$, among other components, in determined concentrations [82,83], which were further demonstrated to interact and generate singlet oxygen, etc. [2,3,13,30]. In addition, appropriate plasma dosing is critical to achieve the desired therapeutic outcome given its dose-dependent nature [84], which has been computationally modeled by us [85].

## 5. Conclusions

Compared with other free radical generating therapies, CAP delivers a mild dose of reactive species, and may provide a safer yet potentially slower alternative for cancer patients unable to tolerate extensive adverse effects associated with conventional approaches. It is worth noting that in cases where ultimate cure is impossible and prolonging life span and enhancing life quality are the primary goals, CAP may be a particularly useful treatment approach. This is because of the limited side effects of reactive species it produces and flexibility of such an approach with respect to treatment delivery. Importantly, cell lines representing triple negative breast cancers that lack clinically useful receptors (estrogen receptor, progesterone receptor, human epidermal growth factor-2) have higher endogenous levels of ROS and are thus more sensitive than their receptor-positive counterparts. These results suggest that CAP may fill the unmet need associated with this breast cancer subtype [50,86]. Synergies between CAP and traditional therapies such as chemotherapy [87] or novel techniques such as iron oxide-based magnetic nanoparticles [88] may increase its efficacy on cancer management. We would foresee CAP being used in a range of contexts. For example, CAP can be used as a clinical tool for surgery such as Canady Helios™ Cold Plasma Scapel (CHCPS) to eradicate residual tumor cells for cancer relapse prevention, as a treatment approach activating the liquid form of chemotherapies for intravenous injection, as an adjuvant drug used, separately, to sensitize tumors developed resistance to existing chemo-, radio-, immuno- or targeted therapies, or as part of a combined modality relying on synergies created between CAP and other chemicals that may or may not convey oncotherapeutics properties by themselves.

Plasma expands new horizons for cell biologists to study cell behavior under extreme stress and mild process environment, often comparable with cancer therapy on patient. The dose-dependent nature of CAP makes it a switch on cancer state control under certain conditions, for example, by inducing cancer cells entering cell cycle arrest or autophagy, CAP may help them surviving redox stress or other unfavorable conditions, despite its selectivity against cancer cells that render it a promising anti-cancer approach. In addition, CAP may potentially be used to model cell transitions from the normal towards cancerous state. Similar to how medical ionizing radiation is used to study exposure of healthy cells to nuclear radiation, exposure to CAP, a unique cocktail of chemical (e.g., ROS, RNS), and physical (e.g., UV and heat) effects can be used to study the synergistic effects of normal environmental conditions that humans encounter daily on natural state transition events during carcinogenesis of, e.g., melanoma. These may represent a novel research avenue in parallel with our current focus on the selectivity of CAP in cancer cells.

## Abbreviations

The following abbreviations are used in this manuscript:

CAP	Cold atmospheric plasma
CHCPS	Canady Helios Cold Plasma Scapel
CSC	Cancer stem cell
IR	Ionizing radiation
PAM	Plasma-activated medium
RONS	Reactive oxygen and nitrogen species
